# Crash causation, countermeasures, and strengthening of road safety policy in Nepal: a narrative review

**DOI:** 10.3389/fpubh.2026.1850342

**Published:** 2026-07-02

**Authors:** Samata Nepal, Nabin Pahari, Purushottam Prasad Yadav, Inesh Khanal, Ritesh G. Menezes, Alok Atreya

**Affiliations:** 1 Lumbini Medical College, Tansen, Nepal; 2 Mercy City Hospital, Rupandehi, Nepal; 3 Provincial Hospital Malangawa, Sarlahi, Nepal; 4 Patan Academy of Health Sciences, Kathmandu, Nepal; 5 Imam Abdulrahman Bin Faisal University College of Medicine, Dammam, Saudi Arabia

**Keywords:** automobiles, distracted driving, driving under the influence, injuries, traffic, pedestrians

## Abstract

Road traffic injuries (RTIs) represent one of the most concerning and unaddressed public health burdens in Nepal, which disproportionately contributes to premature mortality and disability in a country undergoing rapid motorization. Globally RTIs are projected to become one of the top five causes of death by 2030 with low and middle-income countries bearing the greatest burden. This narrative review adopts a multidisciplinary perspective to provide a comprehensive overview of RTIs in Nepal and the measures that can be taken to prevent them. Crash determinants are examined in human, vehicular, and environmental domains, with driver behavior that includes reckless driving, alcohol impairment, and mobile phone use identified as the predominant contributing factor. The review integrates evidence from public health, road engineering, behavioral science, and policy domains to examine the complex determinants of road traffic crashes. It highlights the role of traffic laws and enforcement, infrastructure improvements such as traffic lights and pedestrian crossings, and public education and awareness campaigns in reducing RTIs. Furthermore, the review highlights the urgent need for the Nepalese government to pass the Road Safety Bill, which would empower the National Road Safety Council to implement safer road interventions. By situating Nepal within the broader global context of road safety, this review emphasizes the importance of integrated, multidisciplinary strategies to mitigate RTI and improve outcomes in resource-limited settings.

## 1 Introduction

Traffic-related injuries are a neglected epidemic, not just incidental occurrences (1). They are an inevitable consequence of rapid motorization and urbanization. Road crashes (RC) or road traffic injuries (RTIs) refer to any event involving at least one road user that results in death, injury, or financial loss. According to the global burden of disease study (GBD), RTIs ranked sixth globally in cause -specific mortality in 2023 and are projected to reach the top five by 2030 (2, 3). As of 2022, 56% of the world's population live in urban areas who require vehicles for commuting (4). The increase in vehicle numbers in cities has become unmanageable, increasing the risks of crash, especially for vulnerable road users (VRUs), such as pedestrians, cyclists, and motorcyclists (5).

RTIs account for 1.19 million deaths annually worldwide, and road safety is part of the Sustainable Development Goals, which aim to provide a safe, accessible, affordable and sustainable transportation system by 2030 (6, 7). Of the total annual deaths, 93% occur in LMIC (7). The GBD 2023 study estimated disability adjusted life years for both sexes combined to be 75.3 million for RTI (3). Furthermore, road traffic crashes cost most countries 3% of their gross domestic product (7).

Nepal, like other LMICs, is experiencing rapid motorization. The increasing popularity of motorcycles has correspondingly increased the number of traffic-related injuries and fatalities. Between fiscal years (FY) 2011/12 and 2019/20, RTIs increased from 11,747 to 25,788 (8).

The causes of road crashes are complex and multifactorial. The main contributing factors include speeding, driving under the influence of alcohol, and driver distractions such as using mobile devices while driving or not wearing seatbelts or helmets. Human error is the most common cause of RTI. Other contributing factors include non-compliance with lane markings, improper overtaking, and the desire among vehicles to carry more passengers. Against this backdrop, this narrative review systematically examines the main determinants of road traffic crashes in Nepal, evaluates existing countermeasures, and identifies priority areas for policy strengthening.

## 2 Methods

This narrative review was conducted according to the standard guidelines for narrative reviews on public health topics. A structured search of electronic databases was conducted that included PubMed/MEDLINE, Scopus, and Google Scholar, was performed in August 2025. The search was carried out using the following keywords and their combinations: *road traffic injuries, road traffic accidents, road crashes, Nepal, road safety, crash causation, traffic law enforcement, pedestrian safety*, and *road infrastructure*. Although no formal date restrictions were applied, priority was given to studies published from 2,000 onward to ensure contemporary relevance.

Gray literature was also reviewed to capture relevant policy and surveillance data. The sources included reports and publications from the World Health Organization (WHO), the World Bank, Nepal Police (police mirror), Ministry of Health and Population, the Department of Roads, and Nepal Law Commission.

Articles were included if they (1) addressed road traffic injuries, crash causation or road safety countermeasures; (2) were conducted in Nepal or provided global or regionally generalizable evidence applicable to low- and middle-income countries (LMIC) contexts; and (3) were published in English. Studies were excluded if they were duplicates, lacked sufficient methodological detail, or were not relevant to the scope of this review. The database search initially identified approximately 700 records. After removal of duplicates and title screening, approximately 250 articles remained for abstract review. Four authors independently participated in title and abstract screening. During the initial full text review of the first 30 articles, recurring thematic domains were identified and used to guide narrative synthesis. The included literature was subsequently categorized into major thematic areas that included human behavioral factors, vehicular factors, road infrastructure, environmental factors, and policy related issues. Because many studies addressed overlapping themes, articles with substantially repetitive findings were selectively excluded during synthesis to maintain conceptual clarity and avoid redundancy.

This review adopts a multidisciplinary conceptual framework based on the Haddon matrix, which is a widely used model in road safety research and categorizes crash determinants across three domains: the host (driver/pedestrian behavior), the agent/vehicle (mechanical and structural factors), and the environment (road infrastructure and socio-legal context). These domains interact across three temporal phases: pre-crash, crash, and post-crash, to determine the occurrence and severity of the RTI. In the Nepalese context, this framework is further situated within the broader ‘Safe System approach' which acknowledges that human error is inevitable and therefore the road system must be designed to be forgiving of such errors.

**Figure 1:**
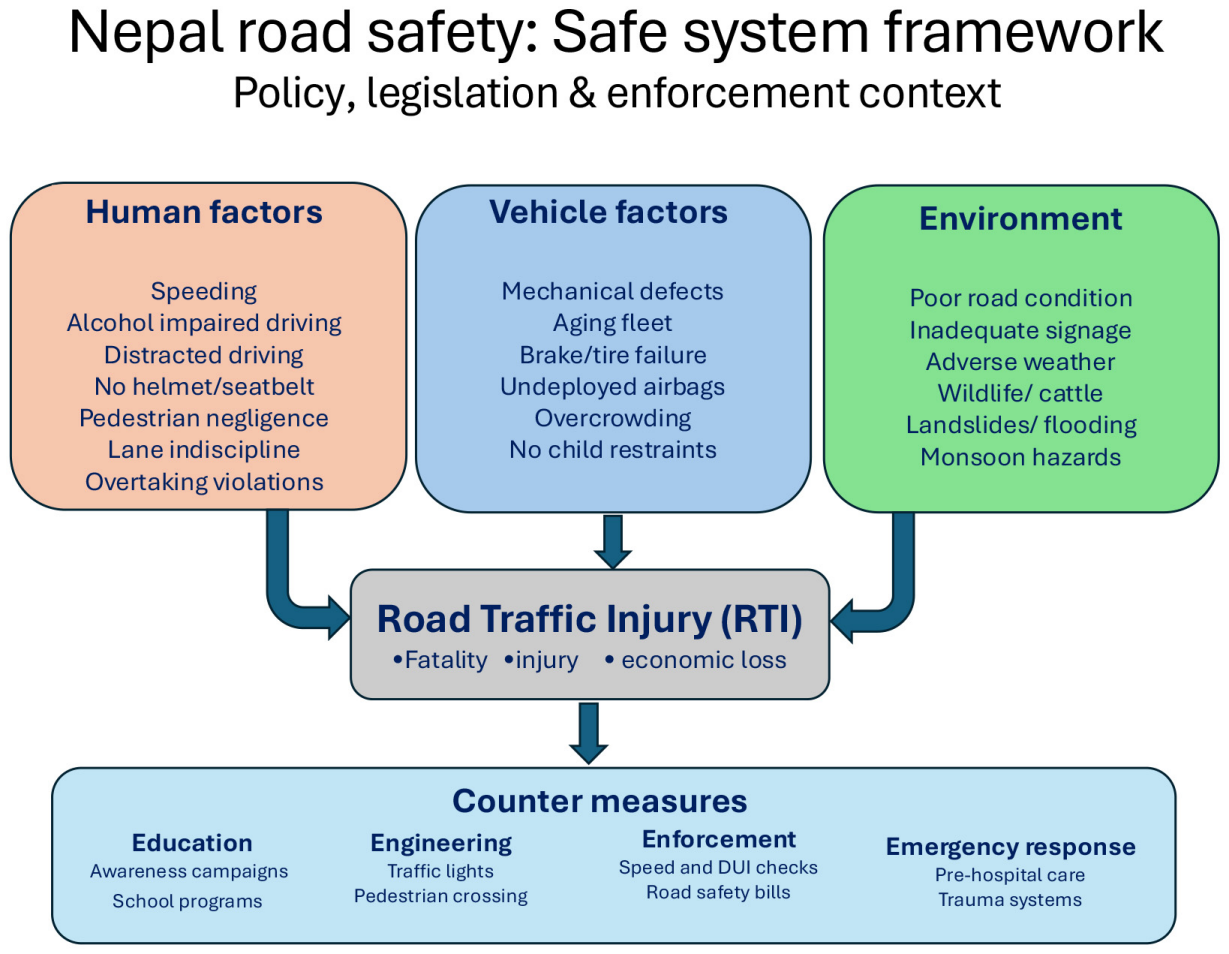
Conceptual framework for the cause of road traffic injury (RTI) and countermeasures in Nepal.

As illustrated in Figure 1, crash causation in Nepal operates through the convergence of human behavioral factors (speeding, alcohol impairment, distraction, non-use of safety gear), vehicular deficiencies (mechanical failures, aging vehicles), and environmental conditions (poor road infrastructure, adverse weather, inadequate signage). These determinants are compounded by systemic gaps in policies and legislative frameworks. Countermeasures that span education, engineering, enforcement, and emergency response are mapped onto these domains to highlight evidence-based intervention points. This integrated framework guides the thematic organization of the present review. This framework also aligns with the principles of the Vision Zero approach, which emphasizes that road traffic deaths and severe injuries are preventable through evidence-based interventions, systems thinking, and shared responsibility among policymakers, road designers, enforcement agencies, vehicle manufacturers, and road users (9). Unlike traditional approaches that primarily attribute crashes to individual error, Vision Zero promotes the development of a safer transport system that anticipates human error and minimizes its harmful consequences (9–11).

## 3 Results and discussion

### 3.1 Overview of road traffic crash trends in Nepal

Nepal has witnessed a sustained and alarming increase in road traffic crashes over the past decade. As shown in Table 1, total crashes increased from 9,145 in 2015 to a peak of 24,537 in 2022, representing a 168% increase over seven years (12). Fatalities similarly increased from 2,004 in 2015 to a peak of 2,883 in 2022 (12). The causes underlying this trend are multifactorial, as detailed in Table 2, with driver negligence accounting for the single largest category of crashes (84,901 over the decade), followed by over-speeding (49,351) and driving under the influence (6,824) (12). This trend emphasizes the urgent need for strengthened road safety policies and regulations, as well as countermeasures to combat crash causation in Nepal.

**Table 1:** Road crashes in Nepal in the past decade (2015–2024) (12).

Year	Total crashes	Injuries	Serious injuries	Fatalities
2015	9,145	8,127	4,054	2,004
2016	10,013	8,219	4,282	2,006
2017	10,178	8,290	4,250	2,385
2018	10,955	8,247	4,144	2,541
2019	13,366	10,360	4,376	2,789
2020	15,559	11,593	4,624	2,251
2021	20,640	18,600	6,448	2,500
2022	24,537	25,722	7,282	2,883
2023	23597	24,488	5,738	2,376
2024	21,287	23,857	5,713	2,198

**Table 2:** Major causes of road traffic crashes in Nepal (2015–2024) (12).

Cause of RTIs	Number of crashes (2015–2024)
Negligence of the driver/rider/Fault of the driver	84,901
Overspeed	49,351
Drink and drive	6,824
The fault of the victim/negligence of pedestrians	6,100
Wrong overtake	3,947
The fault of the vehicle/mechanical defects	3,495
Mobile phone	35
The fault of the animal involved in the incident	593
Bad road conditions	3,247
Environmental factors	383
Overload	411
The fault of another vehicle/human/animal not involved in the incident	Data not available

### 3.2 Negligence of the driver/rider/ fault of the driver

#### 3.2.1 Over speeding

RTIs are significantly affected by speed, since higher speed increases the risk of crashes and makes the consequences more severe (13). The World Health Organization (WHO) states that the likelihood and severity of a crash increase in direct relation to the average increase in the speed of a vehicle (14). Specifically, every 1% increase in mean speed produces a 4% increase in the fatal crash risk and a 3% increase in the serious crash risk (14).

The United Nations hosted the 6th Global Road Safety Week from 17 to 23 May 2021. The goal was to encourage the national and municipal policy to establish and implement 30 km/h speed limits and zones in urban areas, with the support of the local community for low-speed measures.

Over-speeding is the leading contributor to fatal RTIs. Excess speeding reduces reaction time and impairs hazard anticipation (15). Speeding poses a threat not only to the driver's life but also to pedestrians, including law enforcement officers (15). As stated by WHO, the death risk for pedestrians hit by car fronts rises rapidly (4.5 times from 50 km/h to 65 km/h).(14) Excessive speeding, long-distance travel, sleeplessness, and fatigue, followed by negligence or careless driving, are prime causes of RTIs (16, 17).

Young male drivers are more likely to engage in risky behaviors, such as excessive speeding on urban links and driving at night, which increases their risk of severe RTIs and fatalities (17, 18).

In Nepal, there is no specific maximum speed limit designated for vehicles plying on the road. However, for some designated areas, the maximum speed limit is set (Table 3). The absence of a universal national speed limit for general roads in Nepal represents a critical legislative gap. The WHO recommends a maximum speed of 50 km/h on urban roads, reduced to 30 km/h in areas with high concentrations of vulnerable road users such as pedestrians and cyclists. Traffic signals are present where you enter these regions to show the highest legal speed, but they offer no clear instructions on what speed to maintain after passing those initial points. The speed limit signs are worn out and poorly visible to drivers and riders.

**Table 3:** Speed limit regulation in Nepal.

Road type	Speed limit	Enforcement status
Six-lane roads (designated areas)	50 km/h	Limited visibility of signs
School areas	10 km/h	Poor signage
Wildlife reserve highways	10 km/h	Inadequate enforcement
City areas	10–20 km/h	Inconsistent application
General roads	Not specified	No explicit guidance

#### 3.2.2 Driving under the influence

Driving under the influence of alcohol (DUI) is a major traffic problem. Alcohol dependence increases the risk of DUI and contributes to alcohol-related organ complications and withdrawal symptoms. These conditions and hangovers increase the risk of RTI (19). DUI or driving while intoxicated (DWI) involves operating a vehicle with a blood alcohol content (BAC) level of at least 0.08%. The BAC represents the volume of alcohol in the blood and is measured in grams of alcohol per liter of blood (g / l) or its equivalent in exhaled air. Blood alcohol concentration varies with the drinking rate, the type of beverage, food intake, and individual factors such as age, sex, and body weight.

The WHO, in collaboration with the United Nations, launched a global plan for road safety for the decade 2021–2030 to halve the number of alcohol-involved motor vehicle crash injuries and fatalities and reduce the human and economic burden (20). There is a stringent law on drinking and driving in Nepal. The government has imposed zero-level alcohol tolerance in the country since December 2011, and sobriety checkpoints have been established to combat alcohol-impaired driving. This has substantially reduced trauma and traumatic brain injury rates (21). There were 6,824 crashes related to drink and drive in Nepal during the past decade (2015–2024) (Table 4) (12). Although Nepal's legislative stance is stringent, the data in Table 4 show nearly 40,000 cases booked in 2023/24, which highlights that legislation alone is insufficient without consistent and widespread enforcement, particularly outside major urban centers.

**Table 4:** Drink and drive cases in Nepal (12).

Fiscal year^*^	Cases booked	Crashes due to drink and drive
2013/14	61,564	–
2014/15	52,693	–
2015/16	41,720	–
2016/17	38,297	–
2017/18	42,873	288
2018/19	31,978	–
2019/20	‘~40,000	416
2020/21	–	–
2021/22	27,501	–
2022/23	29,597	1,091
2023/24	39,272 (Aug–Jan)	1,211
2024/25		2,050

Empty cell denotes data unavailability.

^*^Nepali fiscal year starts from July 15 each year.

#### 3.2.3 Distractions to the driver

Even minor distractions can cause serious RTIs. They can endanger passengers and others on the road. Mobile phone use; the most common distraction; divides attention and impairs reaction time, contributing to crashes (22). According to the Centers for Disease Control and Prevention (CDC), distractions are of three types: visual, that is, taking eyes off the road, manual, that is, taking hands off the wheel, and cognitive, that is, taking the mind off driving (23). Few examples of driver distractions in Nepal are listed in Table 5. During the last decade, the officially reported mobile phone-related crashes in Nepal remained low, as shown in Table 2. However, mobile phone use while driving is recognized as major source of driver distractions and may be substantially underreported. A study conducted in Israel reported that young drivers with smartphone addiction interacted with their phones an average of 1.71 times per minute while driving, thereby increasing driving risk (24). The widespread availability of ride-sharing and ride-hailing mobile applications may contribute to a reduction in driving under the influence by providing alternative transportation options for the general public in Nepal. Nevertheless, ride-hailing drivers may experience increased fatigue due to frequent travel demands, are mobile phone-related distractions have been associated with a 240% increase in crash risk (25).

**Table 5:** Driver distractions in Nepal.

Distraction type	Examples	Risk level
Visual	Taking eyes off road, billboards, animals	High
Manual	Mobile phone use, adjusting mirrors, eating	High
Cognitive	Conversations, navigation systems, loud music	Medium
Passenger-related	Overcrowding driver cabin, bonnet seating	High
Unreported practices	Conductor conversations while driving	Medium

In developed countries like the United States and England, there are compulsory laws governing front-facing and rear-facing child car seats. However, there is no such rule in Nepal. Children are allowed in the front passenger seat of the car.

Motorcycles are a popular means of transport for low- and middle-income families in Nepal. Motorized two-wheelers in Nepal are two-seater transport. The law is strict in this regard, and the rider will be booked if more than two adult individuals are found riding it, especially in cities where there are traffic police at various checkpoints and road intersections. However, the rule is not followed in rural areas where there are no traffic police. Furthermore, contrary to the law regarding seating capacity for adults, the rule is lenient in the case of children. Parents, along with their children, ride motorcycles without restriction.

#### 3.2.4 Avoiding safety gear like helmets

Nepal is experiencing massive motorization, 77.9% of which is constituted by motorbikes (26). Unlike four-wheelers, motorcycles do not provide cover and protection to their riders and are unstable, making them a dangerous form of transportation. Furthermore, the chance of crashes increases with high speed, poor road conditions, consumption of alcohol or marijuana, and crowded roads (27). Two-wheeler riders primarily comprise people between the ages of 15 and 40. Risk-taking behavior in this age group, with reckless driving, especially among young adults, increases the chances of road mishaps. This includes overtaking in bends, competitive driving behavior, moving forward and filling the gaps in between vehicles, engage in high-risk driving behavior (28).

Helmets are the most effective protection against head and maxillofacial injuries (29). Promoting helmet use while riding two-wheelers is one of the most cost-effective methods to intervene with traffic-related injuries in LMICs, where more than 90% of global traffic-related injuries occur (30). Nepal's law requires mandatory helmet use (with straps on) while riding motorcycles for both the riders and pillion riders; however, the fine is levied only on riders. As the traffic police are lenient toward helmet use for pillion riders, many drivers do not know that this rule exists (31). Furthermore, the law is specific for helmet use, but there is no specification for the type of helmet to be worn. Previous observational studies suggest that helmet selection among some riders may prioritize comfort or aesthetics over certified safety standards (32).

#### 3.2.5 Seatbelts

The most effective way to reduce RTI-induced fatal crashes is by using seatbelts correctly and consistently (33). Victims of serious crash-related injuries are eight times more likely to die if seatbelts are not worn (34). All modern-day vehicles have 3-point seatbelts designed to restrain occupants and lower the risk of being ejected if a crash occurs (34).

Passengers who do not wear seatbelts are most likely to suffer from a frontal crash head injury (34, 35). Although not wearing a seatbelt is against the traffic rule in Nepal, it is not a common practice and is not followed properly. Seatbelt compliance may be motivated primarily by enforcement presence, removing seatbelts once past traffic checkpoints. Furthermore, the seatbelt is buckled behind the back without actually wearing it to stop the alarm. Improperly worn seatbelts cause more harm than good (36, 37). Most modern-day vehicles are designed in a way that if seatbelts are not fastened, airbags won't deploy in case of a serious crash. The reckless behavior of not using seatbelts is mostly seen among young adults (38). There are various factors that affect seatbelt use, e.g., socio-demographic factors, interpersonal and social factors, and environmental factors (39).

#### 3.2.6 Non-adherence to lane driving and overtaking in the wrong manner

Although traffic laws have been strengthened and the traffic police have become more vigilant, the number of RTIs is rising every year. Traffic police cannot possibly monitor every corner, as it is not humanly viable. A lack of lane discipline among drivers means simple overtaking or passing maneuvers are a leading cause of fatal crashes.

Overtaking is a difficult and potentially dangerous maneuver, requiring lane change, lane maintenance, and return to the driving lane, in addition to lateral and longitudinal motion control to prevent collisions (40). A frequent cause of collisions is abrupt lane switching, including overtaking and merging, which has a detrimental effect on the effectiveness of the traffic system (41).

Drivers in Nepal frequently disregard lane discipline. A bold line in the middle of the road means no vehicle is allowed to cross the line, while dotted lines mean that vehicles are allowed to cross the line. Furthermore, there is a lack of courtesy among drivers to let pedestrians cross the road, even at the designated zebra crossings (42).

### 3.3 The fault of the victim/ negligence of pedestrians

Despite the negligence of the drivers, pedestrians are also at fault for causing RTIs. They defy traffic rules and regulations and cross the road carelessly, other than at designated crossings. Walking hurriedly through the road, ignoring the traffic lights against the ‘don't walk' sign, texting or talking on the phone, and not being observant of their surroundings, increases the likelihood of being involved in RTIs.

Zebra crossings are the designated areas for pedestrians to cross the roads. In Nepal, traffic rules are only followed when enforced by the traffic police. The flow of vehicles, especially the motorcycle riders, near zebra crossings at intersections makes it difficult as well as risky for pedestrians. Making the situation much worse, the traffic lights in most places don't work, and the zebra crossings are fading.

A study from the central part of Nepal showed that pedestrians, especially the population of 15–40 years, are the most vulnerable groups in RTIs because pedestrian safety is the least concern for the concerned authority (42). The traffic rule advocates left-side driving and right-side walking so that the pedestrian is aware of the approaching vehicle (42). However, narrow roads, high-beam lights in the late evening (causing sudden and temporary blindness), and perceived pedestrian vulnerability by the approaching vehicle are some of the reasons pedestrians walk in the same direction as the vehicle. Pedestrians need to be visible on the road for their own safety and should avoid wearing dark clothes and use reflectors or reflector-embedded outfits, especially during evening or late evening hours (43). Retro-reflective markings aid in the detection and identification of pedestrians at night, whereas fluorescent colors, notably fluorescent yellow, aid in the visibility and identification of pedestrians to drivers during the day (44).

Pedestrian safety should not be viewed solely as an issue of individual behavior because the built environment strongly influences pedestrian movement and risk exposure (45). Recent urban planning literature emphasizes that pedestrian-centered urban design and improved walkability are essential components of sustainable and safe urban mobility systems (45). Features such as well-maintained sidewalks, visible zebra crossings, adequate street lighting, traffic calming measures, and accessible pedestrian infrastructure encourage safer walking behavior and reduce dependence on motorized transportation (45). Therefore, improving walkability may contribute not only to injury prevention, but also to broader goals of urban sustainability and public health.

### 3.4 The fault of the vehicle/ mechanical defects

In Nepal, mechanical defects of the vehicle contribute to RTIs (Table 6). These defects compromise driver control and safety across multiple vehicle systems. Notably, airbag replacement after deployment is often neglected due to high costs, leaving drivers unprotected in subsequent crashes. To address this, the Government of Nepal should mandate periodic roadworthiness inspections for all registered vehicles, with particular emphasis on critical passive safety components including airbags, seatbelts, and braking systems. Such mandatory vehicle safety inspection programs are well established in high-income countries and have demonstrated effectiveness in reducing mechanically-related crash fatalities. In the Nepalese context, these inspections could be integrated into the existing vehicle renewal and registration process administered by the Department of Transport Management, making compliance administratively feasible without requiring an entirely new regulatory infrastructure. In Nepal, there is no facility for assessing the vehicle's strength; many vehicles are operating in dangerous conditions that increase crash risk. Proper regulations must be put into place to fix the flaws before vehicles are driven on the road.

**Table 6:** Vehicle mechanical defects and their consequences.

Vehicle system	Specific defects	Safety consequences
Tires	Worn-out, underinflated, improperly placed or installed	Loss of balance and vehicle control
Braking system	Damaged pads, lines, cylinders, or pedal	Brake failure leading to unavoidable collision
Engine/transmission	Seized engine or transmission	Sudden loss of power, hazardous situations
Steering system	Power steering pump problems, faulty hydraulic lines	Loss of vehicle control
Visibility system	Faulty wipers during rain, snow, fog	Reduced visibility, difficulty seeing ahead, increased crash risk
Safety equipment	Non-replaced deployed airbags^*^	Lack of protection in subsequent crashes

^*^Airbag replacement is often neglected due to high cost.

In the capital city of Kathmandu, RTIs caused by mechanical defects rose from 101 in the 2011–12 fiscal year to 121 in 2012–13 (46). Consequently, Nepal implemented a ban on automobiles older than 20 years in 2016, which went into effect in March 2018. This measure has demonstrably reduced the incidence of RTIs involving mechanical issues.

### 3.5 The fault of the animal involved in the incident

In Nepal, abandoned cattle are an important but underrecognized contributor to road traffic crashes, particularly along highways and peri-urban areas. Cultural and legal protection surrounding cows; national law (Muluki Ain) prohibits cow slaughter; has contributed to the presence of free-roaming cattle in public spaces. Mechanization of agriculture has reduced the functional use of bulls for plowing, resulting in increasing number of abandoned animals. These animals either enter the city and live in the streets or live on the edge of the jungle. They do not get deeper into the jungle but graze in the vicinity due to fear of wild predators. Many abandoned cattles graze near roadways and highways, increasing the risk of vehicle-animal collisions, particularly at night and in poorly illuminated areas. Animal-related crashes represent an intersection between transportation safety, livestock management, and broader sociocultural practices in Nepal. Stray cattle on highways and urban roads represent a documented road safety hazard in several LMICs. For example, urban cattle roaming has been reported as a growing traffic and sanitation issue in Kenya, where municipalities face increasing incidents involving cattle on roads and associated traffic disruption (47). Similarly, population estimation studies in India demonstrate that large populations of free-roaming cattle exist in urban areas and contribute to road safety risks, particularly in densely populated cities (48).

Highway construction in Nepal often takes the route of forest or conservation areas to avoid land compensation disputes. According to the World Wildlife Fund, highway construction has resulted in an increase in wildlife deaths. Drivers using these roads within the speed limit are also at risk of encountering wildlife. Human-wildlife conflict stands as one of the foremost conservation challenges globally as interactions between humans and wild animals frequently result in conflicts and negative outcomes (49). In Nepal, too, concerns are raised about the need to protect endangered species, lessen conflicts between people and wildlife, and preserve the ecology.

Roadkill is a serious safety issue that claims both human and animal life. Collisions caused by roadkill can result in the death and suffering of animals and/or vehicle occupants struck by vehicles. Animals abandoned on the road can cause vehicle damage, economic losses, and are a distasteful sight for tourists (50).

### 3.6 Bad road conditions

Roads are essential for development, but Nepal faces significant infrastructure challenges that compromise road safety (Table 7). Monsoon rains exacerbate these conditions, creating hazardous travel environments.

**Table 7:** Road infrastructure failures and their impact on road safety.

Infrastructure failure	Safety impact
Unrepaired road surfaces	Vehicle damage, loss of control
Insufficient drainage	Water accumulation, hydroplaning risk
Potholes	Vehicle damage, sudden braking, swerving
Uncovered/poorly maintained manholes	Tire damage, vehicle trapping, collisions
Ditches and sinkholes	Vehicle entrapment, rollover crashes
Monsoon effects (mud, water-filled potholes)	Reduced visibility, loss of traction

The lack of proper discipline in the state's budgetary system, as well as the year-end rush to finish allocated sums for development projects, has contributed to Nepal's poor road infrastructure. In Nepal, as the fiscal year ends during the monsoon season, the government rushes to finish various development projects. This urgent effort includes digging ditches, installing sewerage pipelines, constructing and blacktopping roads, and setting up other essential road infrastructure.

During the first 8 months of the fiscal year 2016–17, only 12,305 kilometers (42.20% of the available road network's 29,157 kilometers) were black-topped. The blacktop road is in disrepair, and no preventative maintenance measures are in place (51). Even though there is no appropriate instruction or signaling for a maximum or minimum speed restriction, Nepalese roadways have speed breakers. Speed breaker signals should be clearly installed in the roadways prior to the speed breaking point to allow the driver and passengers ample time to apply the brakes. However, a crash may occur if a speeding vehicle runs over a bumpy speed breaker due to improper signaling.

### 3.7 Wrong signaling

Traffic control encompasses not only post-mounted signs but also road markings, delineators, road studs, and traffic light signals. Traffic signs are visual road signs that give drivers information using symbols rather than words. The layout of the symbols, the spacing between the letters in both English and Nepali, the colors, etc., must all be considered. It is crucial for drivers who are moving quickly to be able to recognize a sign; thus, the size must also be taken into consideration. At night and from a distance of at least 60 meters (75 meters on national highways), traffic signs must be readily visible.

Traffic signals regulate vehicle flow; their standardized graphic design communicates safety rules across language and literacy barriers. A person must pass the theoretical exam, which covers all traffic signs, in order to pass the driving test.

The different traffic signs or symbols used on Nepali roads help to improve traffic flow and protect lives from the possible risk of RTI by giving drivers the information they need to safely navigate the road ahead.

The Department of Roads in Nepal complies with international standards for traffic lights and road signs. The United Kingdom's practice of driving on the left side of the road has influenced Nepali driving. Caution signs are therefore posted on the left side of the road. For each function, there is a unique group of indicators with a consistent shape. There are three groups:

1. Regulatory signs: These signs issue directives and may be mandatory or prohibitive. The majority of them take the shape of round discs. The ‘Stop' sign and the ‘Give Way' sign, both of which have unique shapes, set these two signs apart from the others.
2. Warning signs: These signs inform drivers of impending risks or possible hazards. These symbols consist of an equilateral triangle with the highest apex.
3. Information signs: These signs provide directions to vehicles. The signs come in many different groups and are all either rectangular or square in design.

Failures in turn signals and faded signals cause collisions and wrecks, which can have harmful or even fatal effects. Traffic lights are signaling equipment found at intersections, zebra crossings, and other locations. To reduce traffic congestion, traffic lights use three colors: red, yellow, and green. The electrical energy needed to power the traffic signals must be constantly supplied because a shortage could cause the lights to go out. Identifying solar energy as a viable option for traffic lights and signals would ensure uninterrupted service.

Misreading intersection signs and failing to notice traffic signals can result in a crash, which can damage the road (52). Roundabouts and intersections with traffic signals or stop signs dramatically reduce injury crashes (53). The Nepali government should intensify its efforts to prevent RTIs by enforcing laws governing speed limits and traffic signals (54).

### 3.8 Environmental factors

A safer road environment is essential to reduce RTI. Nepal's traffic police identify the road sections that are most susceptible to crashes brought on by fog, mist and snowfall, so that drivers can take preventive measures to avoid collisions.

Fog and mist reduce visibility, particularly at blind corners, and increase the crash risk. Fog-related traffic fatalities are on the rise every other year due to various factors, including air pollution. Snow accumulation at various sections of the highways can also impede vehicle flow. Climate change has additional significant effects on road crashes, such as severe and prolonged monsoons and changes in their pattern. Extreme weather conditions such as floods and landslides have a significant impact on Nepal's rural transportation infrastructure, slowing down travel.

Every year, before the rainy season approaches, rock slips and debris falling from numerous highways cause a surge of traffic injuries, particularly along the Mugling-Narayangadh route, one of Nepal's busiest highways. The Mugling-Narayanghad road segment in Nepal experiences a variety of slope failures, including rockslides, rock topples, and debris slide/flow, every year (55).

Heavy monsoon rains in urban areas cause traffic disruptions and increase the possibility of road crashes due to inadequate water drainage and debris control. A study carried out in Pakistan showed that weather conditions such as rain, extreme cold, fog, and heat were all directly associated with the occurrence of road crashes, which is highly relevant to the situation in Nepal (56). Fog, rain, temperature, and other weather-related factors each accounted for 34%, 25%, 21%, and 20% of all traffic incidents, respectively (56).

### 3.9 The fault of another vehicle/human/animal not involved in the incident

Road crashes are not always caused by the vehicles involved in the collision. Drivers may need to apply sudden brakes to avoid pedestrians or animals that cross the road unexpectedly, which can cause the vehicle behind to collide with the stopped vehicle.

The determinants of RTIs identified in Nepal are broadly consistent with findings from other LMIC settings, where risky driving behavior, poor vehicle maintenance, unsafe road infrastructure, and adverse environmental conditions interact to increase crash risk (57). The findings collectively demonstrate that RTIs in Nepal cannot be explained by isolated behavioral failures alone. Rather, crashes emerge from interactions between weak enforcement systems, unsafe infrastructure, rapid urbanization, inadequate public transport alternatives, and risky road-user behavior. This aligns with the Safe System approach, which recognizes that human error is inevitable and that transport systems must therefore minimize the probability that such errors result in fatal or severe injury (58).

## 4 Recommendations

- Awareness campaigns and traffic law education directed at schoolchildren can create responsible individuals for the future. Enforce strict restrictions to ensure that jaywalkers and pedestrians adhere to the law. Integrating road safety education into school curricula, driver licensing programs, and community awareness campaigns may improve long-term compliance with traffic safety practices (59).
- To reduce crash risk associated with distracted driving, enforcement should be combined with behavioral message such as “head up-phone down” approach (60).
- Pedestrian traffic safety is a complex aspect of urban traffic and a significant health determinant associated with the transportation sector. To promote pedestrian safety, central and local governments should provide a need-based infrastructure, especially for vulnerable groups such as the older adult, disabled, and children. Pedestrians should also be educated to be visible on the road by wearing reflectors or bright clothing, and carrying a torch light, especially at night.
- Municipal cattle management programs, designated holding shelters, fencing of high-risk highway corridors, and community-based livestock control measures may reduce animal-related road crashes (61, 62).
- Given Nepal's monsoon vulnerability, rapid response infrastructure (bulldozers, graders, and excavators) should be pre-positioned in landslide-prone areas. Evidence shows that extreme weather conditions (sleet, rain, fog, snow) significantly increase crash frequency through visibility reduction, and infrastructure failure (63).
- In addition to regular sobriety and vehicle registration checks, operational headlights, taillights, and sidelights should also be inspected. During the winter, it should be mandatory to install fog/mist lights.
- The government of Nepal should establish a mandatory periodic vehicle roadworthiness inspection program, integrated into the vehicle registration renewal process. Inspections should cover passive safety systems that should include airbags, seatbelts, and brakes.
- A nationally consistent tiered system with a 50 km/h urban limit, an 80 km/h highway limit, and a 30 km/h limit in school zones, market areas, and wildlife reserve corridors would provide a clear, enforceable standard aligned with international best practice.
- The public awareness campaigns should focus on correcting misconceptions regarding road crash causation while promoting adherence to evidence-based traffic safety measures.

## 5 Conclusions

Policy makers and road safety agencies must urgently address Nepal's increasing burden of RTI. To reduce the number of RTIs and fatalities in Nepal, stringent laws and regulations must be enforced. Additionally, creating awareness videos that educate the public about road safety issues, rules, and potential solutions could be an effective measure.

The driver license require rigorous testing with strict age restrictions enforced. Improving compliance with traffic laws remains essential to reducing RTI burden. The installation of traffic lights and pedestrian crossings throughout the city improves safety for all road users. To reduce road crashes, it is essential to drive carefully, wear seatbelts, stay within the speed limit, stay in your lane, never drink and drive, and wear helmets while riding a bike.

Among the most urgent priorities for Nepal are the enactment of the Road Safety Bill, the implementation of nationally standardized speed limits, the strengthening of traffic law enforcement, the investment in pedestrian-centered infrastructure, and the establishment of mandatory vehicle safety inspections. Given the predominance of preventable behavioral and infrastructural risk factors, a coordinated Safe System approach that integrates engineering, enforcement, education, and emergency response is essential to sustainably reduce RTI-related mortality and disability in Nepal.
